# Supplementary material for: Ex vivo visualization of RNA polymerase III-specific gene activity with electron microscopy
Source: Commun Biol. 2021 Feb 19;4:234. doi: 10.1038/s42003-021-01752-8 (PMC7895982; doi:10.1038/s42003-021-01752-8)
Supplement: Supplementary file 2 — Supplementary Information [file 42003_2021_1752_MOESM2_ESM.pdf]

SUPPLEMENTARY INFORMATION

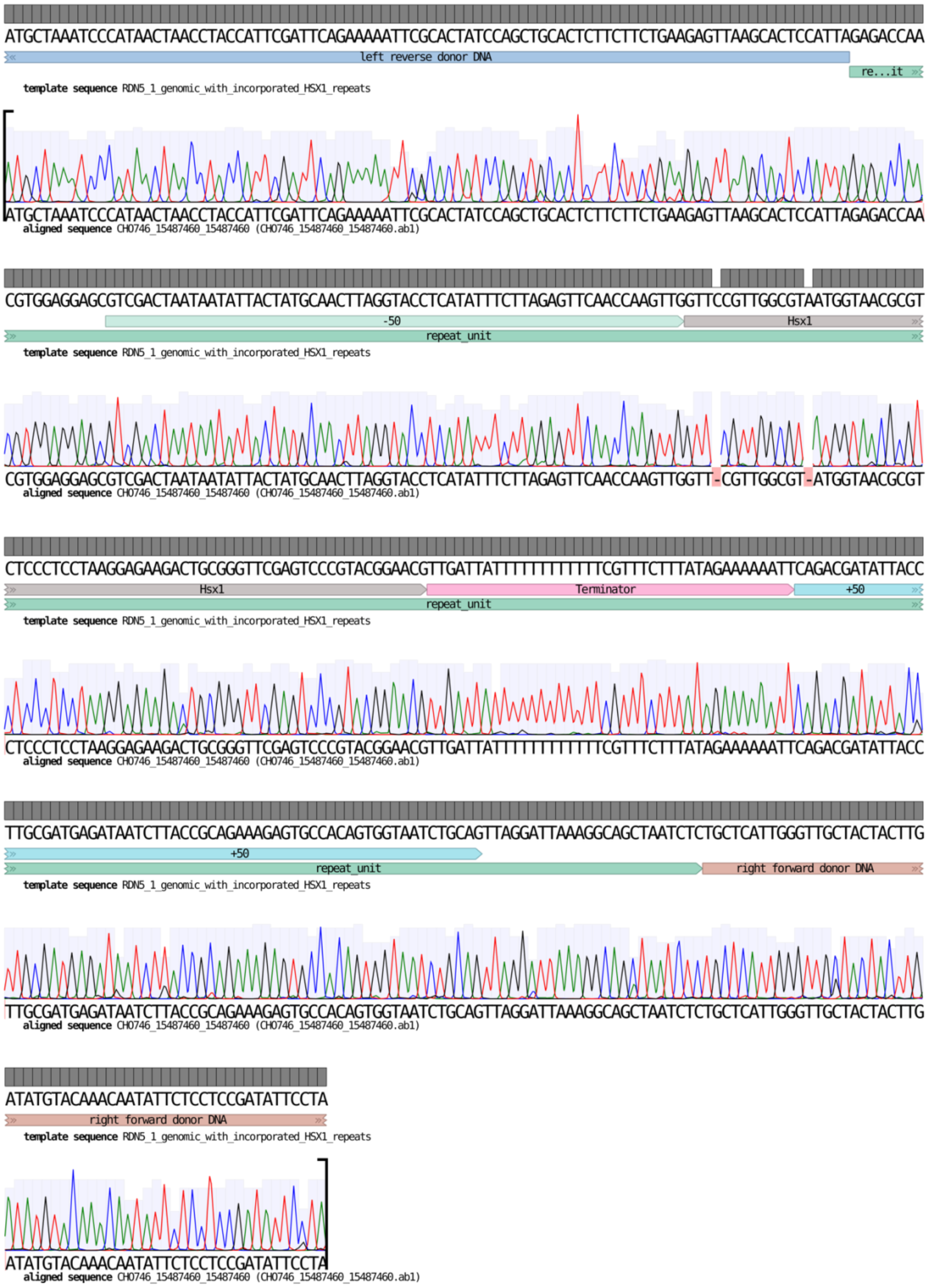

Supplementary Figure 1a: Sanger sequencing results of 5x(O)*RDN5*. The sequence confirms the identity of the *RDN5* copy. It covers the downstream junction between the incorporated copy and the genomic DNA (right forward donor DNA). On the upstream side, parts of another incorporated *RDN5* copy are covered, indicating the incorporation of at least two copies at this genomic site.

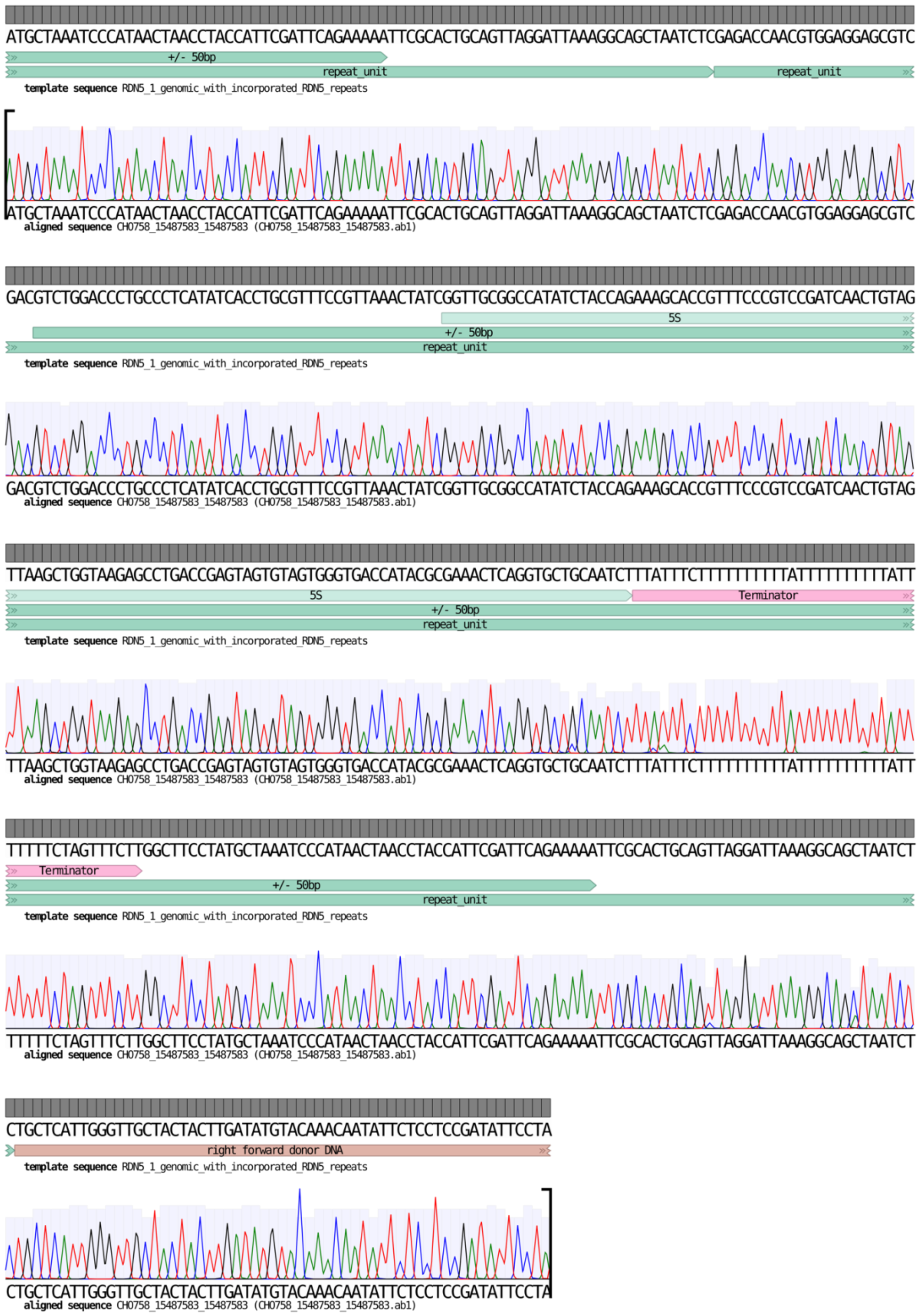

Supplementary Figure 1b: Sanger sequencing results of 2.5x(J)*HSX1*. The sequence confirms the identity of the *HSX1* copy. It covers both the upstream and the downstream junctions between the incorporated copy and the genomic DNA (left reverse donor DNA and right forward donor DNA). At this genomic site, only one copy was incorporated.

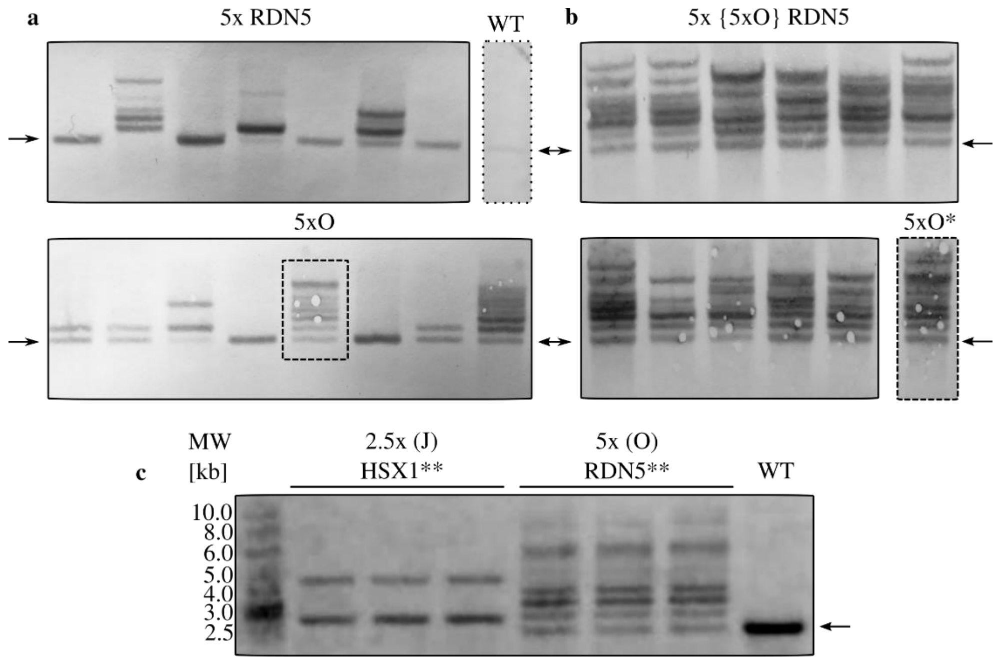

Supplementary Figure 2: Southern blot (SB) analysis of isolated genomic DNA. Dashed arrows mark the bands of the endogenous *RDN5* gene copies and the empty incorporation sites (the endogenous copies are themselves part of the rDNA repeats). **(a) Exemplary SB analysis of clones created in IMX672 (WT, see dotted box) background using editing condition 5x(*RDN5*)** (see Supplementary Table 4)**.** The clone inside the dashed box (5x(O)*RDN5*) was used for further experiments. **(b) SB analysis of iteratively edited and propagated clones**. Clones were created in 5x(O)*RDN5* background using editing condition 5x (see Supplementary Table 4). The clone inside the dashed box (5x(O)*RDN5*) was re-analyzed after three rounds of re-plating (*). **(c) SB analysis of the long-term stability of the clones 2.5x(J)*HSX1* and 5x(O)*RDN5*.** Both clones were re-analyzed after > ten rounds of re-plating over the course of > six month (**) in triplicates from individual plates. We used KPL GeneRuler™ Biotinylatd DNA Ladder. The band pattern of 2.5x(J)*HSX1* perfectly matches that on the SB in Figure 2 (clone from original plate). The band pattern of 5x(O)*RDN5* matches that in Supplementary Figure 2b (after three rounds of re-plating) and Figure 2 (after ~ five rounds of re-plating). In comparison to the band pattern in Supplementary Figure 2a (clone from original plate), additional, higher-migrating bands are visible at the top of the pattern. These could derive from a lower DNA digestion or DNA transfer efficiency of the earliest SB or an early recombination event during strain propagation. As no further changes in band pattern occurred between more often re-plated strains in later SB analyses, we consider the first explanation to be more likely. Later SB analyses (Supplementary Figure 2c and Figure 2) have been performed with digestion and transfer times of > 16h to promote appearance of higher-migrating bands.

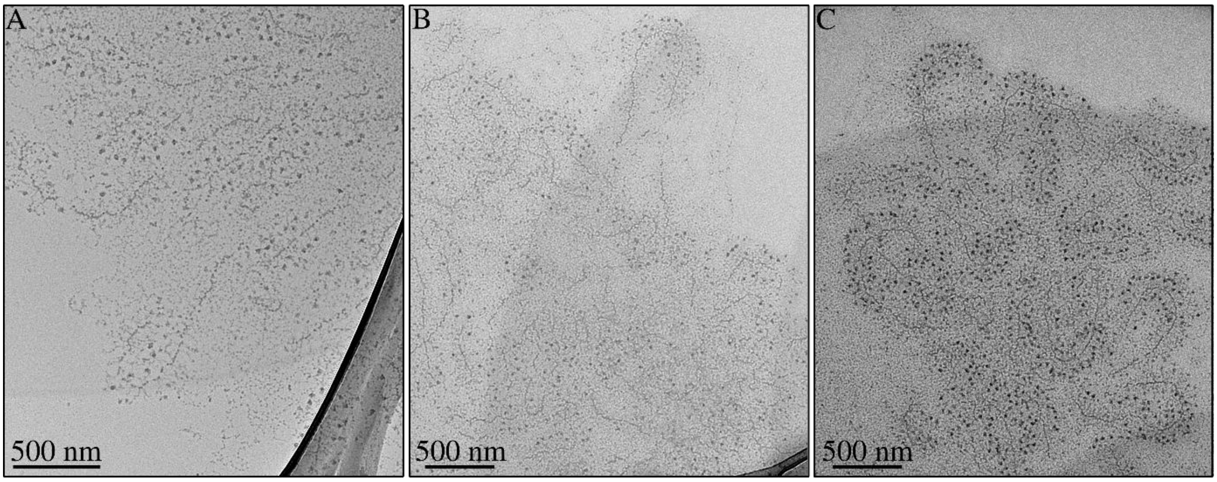

Supplementary Figure 3: Overview of Miller trees in chromatin spreads of (a) IMX672 (WT), (b) 2.5x(J)*HSX1* and (c) 5x(O)*RDN5*. The rDNA repeat seems unchanged in overall appearance and numbers of Miller trees. The samples were positively stained with UA and PTA; EM images were acquired at 12,000x magnification at a defocus of –40 µm.

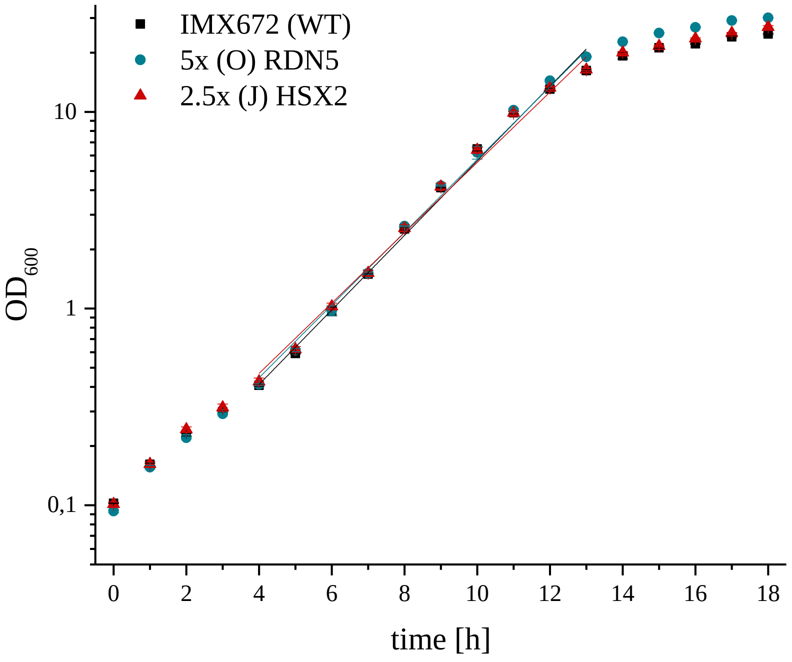

Supplementary Figure 4: Growth of IMX672 (WT), 5x(O)*RDN5* and 2.5x(J)*HSX1*. The OD_{600} of the strains were measured every hour over the course of 18 hours for three distinct replicates per strain. The curves were analyzed by logarithmic transformation and linear fit from t = 3 to t = 13 (exponential growth phase) with line slopes being 0.189 ± 0.006 (R^2 = 0.992) for IMX672 (WT), 0.185 ± 0.004 (R^2 = 0.996) for 5x(O)*RDN5* and 0.179 ± 0.006 (R^2 = 0.994) for 2.5x(J)*HSX1*. Error bars represent standard deviations.

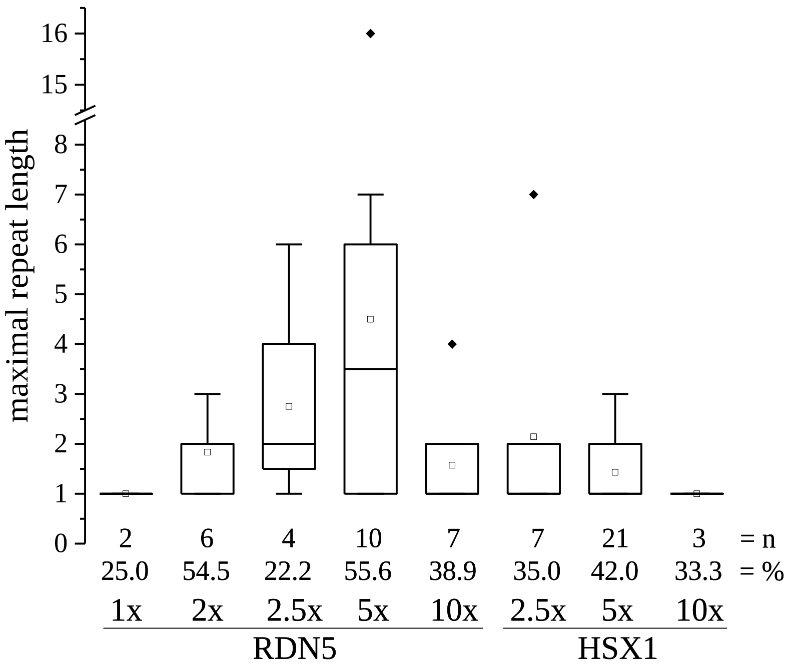

Supplementary Figure 5: Boxplot analysis of maximal incorporated copy numbers for all experimental conditions. Box = 25–75%, bracket = range within 1.5 IQR, horizontal line = median, square = mean, diamonds = outliers. Boxplots include only clones with a positive incorporation result. The number of positive clones and their percentage in relation to all analyzed clones are indicted below the boxes. Sample-sizes were based on the maximal number of samples that could be analyzed in parallel by Southern blotting. No data was excluded, all analyzed clones were included in the box plot.

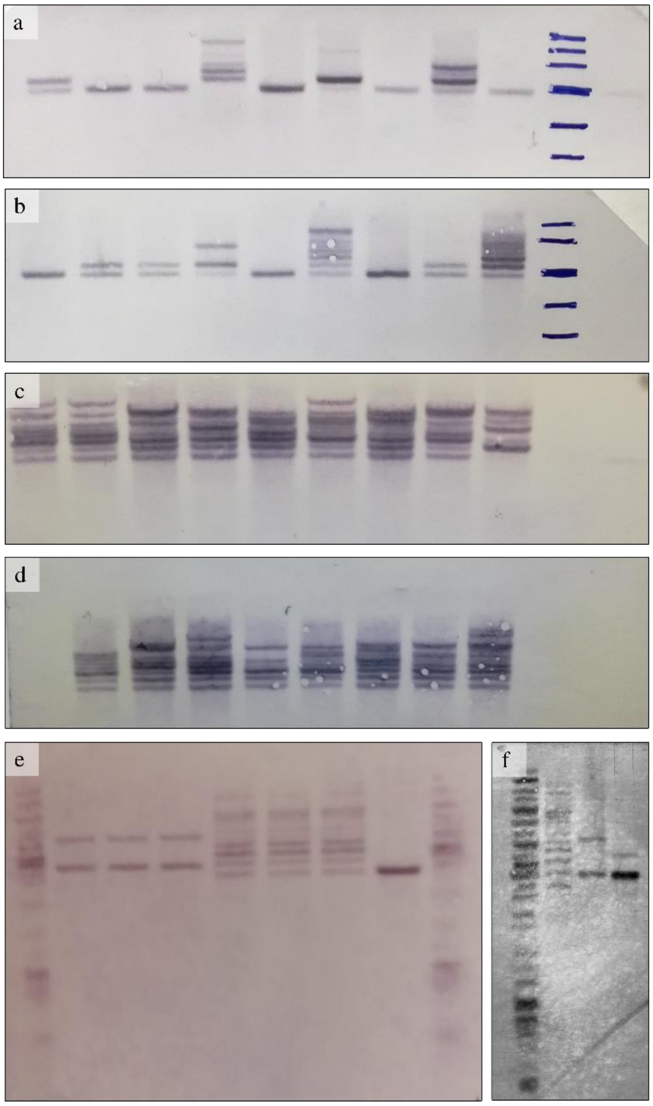

Supplementary Figure 6: Raw and uncropped Southern blot membranes. (a - b) Supplementary Figure 2a (c - d) Supplementary Figure 2b (e) Supplementary Figure 2c (f) Figure 2a

Supplementary Table 1: Sequences of the oligonucleotides used to assemble the repeat unit.

	Sequence 5’ to 3’
Oligo I (fw) RDN5	GAGACCAACGTGGAGGAGCGTCGACGTCTGGACCCTGCCCTCATATCACCTG
Oligo II (rev) RDN5	CCCTGCCCTCATATCACCTGCGTTTCCGTTAAACTATCGGTTGCGGCCATAT
Oligo III (fw) RDN5	ACTATCGGTTGCGGCCATATCTACCAGAAAGCACCGTTTCCCGTCCGATCAA
Oligo IV (rev) RDN5	ACCGTTTCCCGTCCGATCAACTGTAGTTAAGCTGGTAAGAGCCTGACCGAGT
Oligo V (fw) RDN5	TGGTAAGAGCCTGACCGAGTAGTGTAGTGGGTGACCATACGCGAAACTCAGG
Oligo VI (rev) RDN5	GACCATACGCGAAACTCAGGTGCTGCAATCTTTATTTCTTTTTTTTTTATTT
Oligo VII (fw) RDN5	TATTTCTTTTTTTTTTATTTTTTTTTTATTTTTTTCTAGTTTCTTGGCTTCC
Oligo VIII (rev) RDN5	TTTCTAGTTTCTTGGCTTCCTATGCTAAATCCCATAACTAACCTACCATTCGATTCAGAA
Oligo IX (fw) RDN5	CATAACTAACCTACCATTCGATTCAGAAAAATTCGCACTGCAGTTAGGATTA
Oligo X (rev) RDN5	CGCTCCTCCACGTTGGTCTCGAGATTAGCTGCCTTTAATCCTAACTGCAGTGCGA
Oligo I (fw) HSX1	GAGACCAACGTGGAGGAGCGTCGACTAATAATATTACTATGCAACTTAGG
Oligo II (rev) HSX1	CCAACTTGGTTGAACTCTAAGAAATATGAGGTACCTAAGTTGCATAGTAATA
Oligo III (fw) HSX1	TAGAGTTCAACCAAGTTGGTTCCGTTGGCGTAATGGTAACGCGTCTCCCTCC
Oligo IV (rev) HSX1	CGTACGGGACTCGAACCCGCAGTCTTCTCCTTAGGAGGGAGACGCGTTACCA
Oligo V (fw) HSX1	CGGGTTCGAGTCCCGTACGGAACGTTGATTATTTTTTTTTTTTCGTTTCTTTATA
Oligo VI (rev) HSX1	GATTATCTCATCGCAAGGTAATATCGTCTGAATTTTTTCTATAAAGAAACGAAAAAAA
Oligo VII (fw) HSX1	ACCTTGCGATGAGATAATCTTACCGCAGAAAGAGTGCCACAGTGGTAATCTGCAGTTA
Oligo VIII (rev) HSX1	GCTCCTCCACGTTGGTCTCGAGATTAGCTGCCTTTAATCCTAACTGCAGATTACCACTG

Supplementary Table 2: Sequences of the donor DNA pieces.

	Sequence 5’ to 3’
Left reverse donor DNA RDN5	**TCCAGACGTCGACGCTCCTCCACGTTGGTCTC**TAATGGAGTGCTTAACTCTTCAGAAGAAGAGTGCAGCTGGATAGTGCGAATTTTTCTGAATCGAATGGTAGGTTAGTTATGGGATTTAGCATAGGAAGCCAAGAAACTAGAAAAAAAAAAAAAAAAAAAAAAAAAAAAAGAAATAAAGATTGCAGCACCTGAGTTTCGCGTATGGTCACCCACTACACTACTCGGTCAGG<br>**Reverse begin of repeat** + reverse 200 bp genomic DNA

Right forward donor DNA RDN5	**CTACCATTCGATTCAGAAAAATTCGCACTGCAGTTAGGATTAAAGGCAGCTAATCTC**TGCTCATTGGGTTGCTACTACTTGATATGTACAAACAATATTCTCCTCCGATATTCCTACAAAAAAAAAAAAAAAAACACTCCGGTTTTGTTCTCTTCCCTCCATTTCCCTCTCTTCTACGGTTAATACTTTCCTCTTCGTCTTTTTCTACACCCTCGTTTAGTTGCTTCTTATTCCTTCCCGCTTTCCTGCACTAACAT
	**Forward end of repeat** + forward 200 bp genomic DNA
Long left reverse donor DNA RDN5	**TCCAGACGTCGACGCTCCTCCACGTTGGTCTC**TAATGGAGTGCTTAACTCTTCAGAAGAAGAGTGCAGCTGGATAGTGCGAATTTTTCTGAATCGAATGGTAGGTTAGTTATGGGATTTAGCATAGGAAGCCAAGAAACTAGAAAAAAAAAAAAAAAAAAAAAAAAAAAAAGAAATAAAGATTGCAGCACCTGAGTTTCGCGTATGGTCACCCACTACACTACTCGGTCAGGCTCTTACCAGCTTAACTACAGTTGATCGGACGGGAAACGGTGCTTTCTGGTAGATATGGCCGCAACCGATAGTTTAACGGAAACGCAGGTGATATGAGGGCAGGGTCCAGACATGTTCAGTAGGTGGGAGTGAGAGGTGTTATGGGTGGAGGACAATTTTTATTATATTTCATCTAATAGCAATAGGATATGACAGGTGAAAAAGCAAAAGCAATAGTGCATTGTGATGTGGAGAATAAGGTGCATACGATGAAAAAGGTGATTTGTCATTTACAAGAGGTAGGTCGAAACAGAACATGAAAGTTGGTCGGTAGGTGGCATGCAGAGGTAGTTTCAAGGTGACAGGTTATGAAGATATGGTGCAAAAGACAAATGGATGGTGGCAGGCATAGTAAAATGATGGTGTGGAAGACATAGATGGTATTTGTTTTGCATTTACGGCACCGGATGCGGGCGATAATGACGGGAAGAGATTTAGTATGTGGGACAGAATGTCGGCGGCAGTATTGAGACCATGAGAGTAGCAAACGTAAGTCTAAAGGTTGTTTTATAGTAGTTAGGATGTAGAAAATGTATTCCGATAGGCCATTTTACATTTGGAGGGACGG
	**Reverse begin of repeat** + reverse 808 bp genomic DNA
Long right forward donor DNA RDN5	**CTACCATTCGATTCAGAAAAATTCGCACTGCAGTTAGGATTAAAGGCAGCTAATCTC**TGCTCATTGGGTTGCTACTACTTGATATGTACAAACAATATTCTCCTCCGATATTCCTACAAAAAAAAAAAAAAAAACACTCCGGTTTTGTTCTCTTCCCTCCATTTCCCTCTCTTCTACGGTTAATACTTTCCTCTTCGTCTTTTTCTACACCCTCGTTTAGTTGCTTCTTATTCCTTCCCGCTTTCCTGCACTAACATTTTGCCGCATTACACTATATGATCGTAGTACATCTTACAACTCCGCATACCGCGTCGCCGCGTCGCCGCGTCGCCAAAAATTTACTTCGCCAACCATTCCATATCTGTTAAGTATACATGTATATATTGCACTGGCTATTCATCTTGCACTTTTCCTCTTTCTTCTTCCCAGTAGCCTCATCCTTTTACGCTGCCTCTCTGGAACTTGCCATCATCATTCCCTAGAAACTGCCATTTACTTAAAAAAAAAAAAAAAAAAAAAATGTCCCCACTGTTCACTGTTCACTGTTCACTTGTCTCTTACATCTTTCTTGGTAAAATCGTAGTTCGTAGTATTTTTTTTCATATCAAAGGCATGTCCTGTTAACTATAGGAAATGAGCTTTTCTCAATTCTCTAAACTTATACAAGCACTCATGTTTGCCGCTCTGATGGTGCGGAAAAAACTGCTCCATGAAGCAAACTGTCCGGGCAAATCCTTTCACGCTCGGGAAGCTTTGTGAAAGCCCTTCTCTTTCAACCCATCTTTGCAACGAAAAAAAAAAAAAAAAATAAAAAATAAAAAGACCAAATAGTAAATAGTAACTTACATACATTAGTAAATGGTACACTCTTACACACTATCATCCTCATCG
	**Forward end of repeat** + forward 833 bp genomic DNA
Long left reverse donor DNA HSX1	**GCATAGTAATATTATTAGTCGACGCTCCTCCACGTTGGTCTC**TAATGGAGTGCTTAACTCTTCAGAAGAAGAGTGCAGCTGGATAGTGCGAATTTTTCTGAATCGAATGGTAGGTTAGTTATGGGATTTAGCATAGGAAGCCAAGAAACTAGAAAAAAAAAAAAAAAAAAAAAAAAAAAAAGAAATAAAGATTGCAGCACCTGAGTTTCGCGTATGGTCACCCACTACACTACTCGGTCAGGCTCTTACCAGCTTAACTACAGTTGATCGGACGGGAAACGGTGCTTTCTGGTAGATATGGCCGCAACCGATAGTTTAACGGAAACGCAGGTGATATGAGGGCAGGGTCCAGACATGTTCAGTAGGTGGGAGTGAGAGGTGTTATGGGTGGAGGACAATTTTTATTATATTTCATCTAATAGCAATAGGATATGACAGGTGAAAAAGCAAAA

	GCAATAGTGCATTGTGATGTGGAGAATAAGGTGCATACGATGAAAAAGGT GATTTGTCATTTACAAGAGGTAGGTCGAAACAGAACATGAAAGTTGGTCG GTAGGTGGCATGCAGAGGTAGTTTCAAGGTGACAGGTTATGAAGATATGG TGCAAAAGACAAATGGATGGTGGCAGGCATAGTAAAATGATGGTGTGGAA GACATAGATGGTATTTGTTTTGCATTTACGGCACCGGATGCGGGCGATAAT GACGGGAAGAGATTTAGTATGTGGGACAGAATGTCGGCGGCAGTATTGAG ACCATGAGAGTAGCAAACGTAAGTCTAAAGGTTGTTTTATAGTAGTTAGGA TGTAGAAAATGTATTCCGATAGGCCATTTTACATTTGGAGGGACGG<br>**Reverse begin of repeat** + reverse 808 bp genomic DNA
Long right forward donor DNA HSX1	**CAGTGGTAATCTGCAGTTAGGATTAAAGGCAGCTAATCTC**TGCTCATTG GGTTGCTACTACTTGATATGTACAAACAATATTCTCCTCCGATATTCCTACA AAAAAAAAAAAAAAAACACTCCGGTTTTGTTCTCTTCCCTCCATTTCCCT CTCTTCTACGGTTAATACTTTCCTCTTCGTCTTTTTCTACACCCTCGTTTAGT TGCTTCTTATTCCTTCCCGCTTTCCTGCACTAACATTTTGCCGCATTACACTA TATGATCGTAGTACATCTTACAACTCCGCATACCGCGTCGCCGCGTCGCCG CGTCGCCAAAAATTTACTTCGCCAACCATTCCATATCTGTTAAGTATACATG TATATATTGCACTGGCTATTCATCTTGCACTTTTCCTCTTTCTTCTTCCCAGT AGCCTCATCCTTTTACGCTGCCTCTCTGGAACTTGCCATCATCATTCCCTAG AAACTGCCATTTACTTAAAAAAAAAAAAAAAAAAAAAAATGTCCCCACTGT TCACTGTTCACTGTTCACTTGTCTCTTACATCTTTCTTGGTAAAATCGTAGT TCGTAGTATTTTTTTTCATATCAAAGGCATGTCCTGTTAACTATAGGAAATG AGCTTTTCTCAATTCTCTAAACTTATACAAGCACTCATGTTTGCCGCTCTGA TGGTGCGGAAAAAACTGCTCCATGAAGCAAACTGTCCGGGCAAATCCTTT CACGCTCGGGAAGCTTTGTGAAAGCCCTTCTCTTTCAACCCATCTTTGCAA CGAAAAAAAAAAAAAAAATAAAAAATAAAAAGACCAAATAGTAAATAGT AACTTACATACATTAGTAAATGGTACACTCTTACACACTATCATCCTCATCG<br>**Forward end of repeat** + forward 833 bp genomic DNA

Supplementary Table 3: Sequences of all primers used.

Abbreviation	Function	Sequence
p_1.1_fw	pMEL10 linearization	GTTTTAGAGCTAGAAATAGCAAGTTAAAATAAGGCTAG TC
p_1.2_rev	pMEL10 linearization	GATCATTTATCTTTCACTGCGGAGAAG
p_2.1_fw	left 70 bp gRNA cassette	TGCGCATGTTTCGGCGTTCGAAACTTCTCCGCAGTGAA AGATAAATGATCTAGCAACCCAATGAGCATAA
p_2.2_rev	right 70 bp gRNA cassette	GTTGATAACGGACTAGCCTTATTTTAACTTGCTATTTCTA GCTCTAAAACTTATGCTCATTGGGTTGCTA
p_3.1_fw	left reverse donor RDN5	TCCAGACGTCGACGCTCCTCCACGTTGGTCTCTAATGG AGTGCTTAACTCTTCAGAAGAAG
p_3.2_rev	left reverse donor RDN5	CCTGACCGAGTAGTGTAGTGGGTG
p_3.3_fw	right forward donor RDN5	CTACCATTCGATTCAGAAAAATTCGCACTGCAGTTAGGA TTAAAGGCAGCTAATCTCTGCTCATTGGGTTGCTACTAC TTG
p_3.4_rev	right forward donor RDN5	ATGTTAGTGCAGGAAAGCGGGAAG
p_3.5_fw	long left reverse donor RDN5	TCCAGACGTCGACGCTCCTCCACGTTGGTCTCTAATGG AGTGCTTAACTCTTCAGAAGAAG
p_3.6_rev	long left reverse donor RDN5/HSX1	CCGTCCCTCCAAATGTAAAATGGCCTATCGG

p_3.7_fw	long right forward donor RDN5	CTACCATTCGATTCAGAAAAATTCGCACTGCAGTTAGGATTAAAGGCAGCTAATCTCTGCTCATTGGGTTGCTACTACTTG
p_3.8_rev	long right forward donor RDN5/HSX1	CGATGAGGATGATAGTGTGTAAGAGTGTACC
p_3.9_fw	long left reverse donor HSX1	GCATAGTAATATTATTAGTCGACGCTCCTCCACGTTGGTCTCTAATGGAGTGCTTAACTCTTCAGAAGAAG
p_3.10_fw	long right forward donor HSX1	CAGTGGTAATCTGCAGTTAGGATTAAAGGCAGCTAATCTCTGCTCATTGGGTTGCTACTACTTG
p_5.1_fw	Pol3 RDN5/HSX1 repeat unit	GAGACCAACGTGGAGGAGC
p_5.2_rev	Pol3 RDN5/HSX1 repeat unit	GAGATTAGCTGCCTTTAATCCTAA
p_6.1_fw	p414-TEF1p-Cas9-CYC1t linearization	TCTCAGCTCGGTGGAGACAGCAG
p_6.2_rev	p414-TEF1p-Cas9-CYC1t linearization	TTTCCCGGGGGATCCACTAGTTCTAG
p_6.3_fw	PZF1 insert generation	CTAATCTAAGTTTTCTAGAACTAGTGGATCCCCCGGGAAAAGTAACTGTTGAAATCGCTGTC
p_6.4_rev	PZF1 insert generation	GCGTGACATAACTAATTACATGACTCGAGGTCGACACCAATTGCAGTAACAAAATGGC

Supplementary Table 4: Amounts of components used to transform IMX672 for CRISPR/Cas9 incorporation experiments.

	RDN5					HSX1		
	1x	2x	2.5x	5x	10x	2.5x	5x	10x
pMEL10_ gRNA [µg]	1	2	1	5	5	1	2.5	2.5
Right_fw donor DNA [pmol]	5	10	5	14.5	16.2	6.3	11.5	9.8
Left_rev donor DNA [pmol]	5	10	5	14.5	16.2	6.3	11.5	9.8
Long right_fw donor DNA [pmol]	-			3.2	5.8	3.9	5.0	4.2
Long left_rev donor DNA [pmol]	-			3.2	5.8	3.9	5.0	4.2
Oligonucleotides (each) [pmol]	100	200	250	500	1000	250	500	1000

Supplementary Table 5: Numbers of Pol III complexes over all analyzed pairs of Miller trees from 5x(O)RDN5, 5x(O)RDN5 with TFIIIA overexpression and 2.5x(J)HSX1.

	Trees per strain			
Pol III complexes	5x(O)*RDN5*	5x(O)*RDN5* with TFIIIA	2.5x(J)*HSX1*	
0	10	3	6	
1	0	1	3	
2	4	7	5	
3	6	3	5	
4	7	10	5	
5	3	10	6	
6	2	7	3	
7	1	4	0	
8	1	8	1	
9	1	0	1	
10	0	1	0	
	35	48	41	= n
