# Supplementary material for: Ex vivo visualization of RNA polymerase III-specific gene activity with electron microscopy
Source: Commun Biol. 2021 Feb 19;4:234. doi: 10.1038/s42003-021-01752-8 (PMC7895982; doi:10.1038/s42003-021-01752-8)
Supplement: Supplementary file 3 — Reporting Summary [file 42003_2021_1752_MOESM3_ESM.pdf]

nature research

Corresponding author(s): Achilleas Frangakis

Last updated by author(s): Dec 7, 2020

Reporting Summary

Nature Research wishes to improve the reproducibility of the work that we publish. This form provides structure for consistency and transparency in reporting. For further information on Nature Research policies, see our Editorial Policies and the Editorial Policy Checklist.

Statistics

For all statistical analyses, confirm that the following items are present in the figure legend, table legend, main text, or Methods section.

n/a	Confirmed	
☐	☒	The exact sample size (*n*) for each experimental group/condition, given as a discrete number and unit of measurement
☐	☒	A statement on whether measurements were taken from distinct samples or whether the same sample was measured repeatedly
☒	☐	The statistical test(s) used AND whether they are one- or two-sided<br>*Only common tests should be described solely by name; describe more complex techniques in the Methods section.*
☒	☐	A description of all covariates tested
☒	☐	A description of any assumptions or corrections, such as tests of normality and adjustment for multiple comparisons
☒	☐	A full description of the statistical parameters including central tendency (e.g. means) or other basic estimates (e.g. regression coefficient) AND variation (e.g. standard deviation) or associated estimates of uncertainty (e.g. confidence intervals)
☒	☐	For null hypothesis testing, the test statistic (e.g. *F*, *t*, *r*) with confidence intervals, effect sizes, degrees of freedom and *P* value noted<br>*Give P values as exact values whenever suitable.*
☒	☐	For Bayesian analysis, information on the choice of priors and Markov chain Monte Carlo settings
☒	☐	For hierarchical and complex designs, identification of the appropriate level for tests and full reporting of outcomes
☒	☐	Estimates of effect sizes (e.g. Cohen's *d*, Pearson's *r*), indicating how they were calculated

Our web collection on statistics for biologists contains articles on many of the points above.

Software and code

Policy information about availability of computer code

Data collection	Image aquisition on Tecnai F30 G2 transmission electron microscope: Thermo Fisher Scientific software TIA (TEM Imaging & Analysis version 4.15 SP1) or EPU (version 1.11.1.50REL)<br>Image aquisition on Titan Krios transmission electron microscope: SerialEM v3.8.0beta
Data analysis	Image display software: Fiji/ImageJ

For manuscripts utilizing custom algorithms or software that are central to the research but not yet described in published literature, software must be made available to editors and reviewers. We strongly encourage code deposition in a community repository (e.g. GitHub). See the Nature Research guidelines for submitting code & software for further information.

Data

Policy information about availability of data

All manuscripts must include a data availability statement. This statement should provide the following information, where applicable:

- Accession codes, unique identifiers, or web links for publicly available datasets
- A list of figures that have associated raw data
- A description of any restrictions on data availability

The data that support the findings of this study are available on request from the corresponding author.

Field-specific reporting

Please select the one below that is the best fit for your research. If you are not sure, read the appropriate sections before making your selection.

☒ Life sciences ☐ Behavioural & social sciences ☐ Ecological, evolutionary & environmental sciences

For a reference copy of the document with all sections, see nature.com/documents/nr-reporting-summary-flat.pdf

Life sciences study design

All studies must disclose on these points even when the disclosure is negative.

Sample size	I. Figure A5: Sample-sizes were based on the maximal number of samples that could be analyzed in parallel by Southern Blotting.<br>II. Figure 4: Sample-sizes were chosen to be >30 to allow proper comparability in a box plot and limited by the acquisition capacities of two microscope sessions.
Data exclusions	I. No data was excluded, all analyzed clones were included in the box plots.<br>II. All acquired images with well-spread Miller trees were included in the analysis.
Replication	I. Reproducibility (here: genomic stability of the clones) was confirmed by Southern blot analysis before and after repetitive re-plating of two exemplary clone. The analysis after propagation was done in triplicates.<br>II. Images of two independent preparations of chromatin based off two different plates were included in the analysis.<br>III. Growth curves: All strains were analysed in distinct triplicates.
Randomization	Not relevant
Blinding	Not relevant

Reporting for specific materials, systems and methods

We require information from authors about some types of materials, experimental systems and methods used in many studies. Here, indicate whether each material, system or method listed is relevant to your study. If you are not sure if a list item applies to your research, read the appropriate section before selecting a response.

Materials & experimental systems

n/a	Involved in the study	
☒	☐	Antibodies
☐	☒	Eukaryotic cell lines
☒	☐	Palaeontology and archaeology
☒	☐	Animals and other organisms
☒	☐	Human research participants
☒	☐	Clinical data
☒	☐	Dual use research of concern

Methods

n/a	Involved in the study	
☒	☐	ChIP-seq
☒	☐	Flow cytometry
☒	☐	MRI-based neuroimaging

Eukaryotic cell lines

Policy information about cell lines

Cell line source(s)	S. cerevisiae strain IMX672 (Euroscarf, Oberursel, Germany, #Y40595) was used as a parental strain.
Authentication	Authentication of the modified genome areas by Southern blotting and Sanger sequencing.
Mycoplasma contamination	The yeast strains were not tested for Mycoplasma contamination.
Commonly misidentified lines<br>(See ICLAC register)	*Name any commonly misidentified cell lines used in the study and provide a rationale for their use.*
